# Maintaining Oxygenation Successfully with High Flow Nasal Cannula during Diagnostic Bronchoscopy on a Postoperative Lung Transplant Patient in the Intensive Care

**DOI:** 10.1155/2014/198262

**Published:** 2014-11-13

**Authors:** Sara Diab, John F. Fraser

**Affiliations:** Critical Care Research Group, Adult Intensive Care Service, The Prince Charles Hospital, Brisbane, QLD 4032, Australia

## Abstract

Bronchoscopy is an important diagnostic and therapeutic intervention for a variety of patients displaying pulmonary pathology. The heterogeneity of the patients undergoing bronchoscopy affords a challenge for providing minimal and safe respiratory support during anesthesia. Currently, options are intubation and general anesthesia versus frequently inadequate sedation or local anaesthesia with low flow oxygen through nasal prongs or mouthpiece. The advent of high flow nasal cannula allows the clinician to have a “middle man” that allows high flow oxygen delivery as well as a degree of respiratory support, which in some cases has been noted to be between 3 and 4 cm of continuous positive airway pressure-like effect. There are minimal data analyzing the use of high flow nasal cannula during anesthesia for bronchoscopy. We present a case report of orthotropic lung transplant recipient undergoing diagnostic bronchoscopy whilst being supported with high flow nasal oxygen in the intensive care unit.

## 1. Introduction

Bronchoscopy is an invasive procedure, which occurs in patients with some degree of pulmonary pathology, performed for diagnostic and therapeutic intervention. Induction of general anesthesia for bronchoscopy is associated with certain risks including hypoxemia, increased work of breathing, collapse of upper airways, and reduction in end-expiratory lung volumes. Noninvasive ventilation (NIV) has been widely evaluated for safe use during bronchoscopy by way of facemask with continuous positive airway pressure delivered [1]. High flow nasal cannula (HFNC) oxygenation is an emerging therapy for respiratory support that delivers heated and humidified air and oxygen with flows of up to 60 L/min. Diverse capability for effective therapeutic use of HFNC is growing in the literature, with the presence of potentially beneficial pharyngeal positive pressure repeatedly reported [2–5]. This case report demonstrates the successful maintenance of oxygenation on a postoperative lung transplant subject receiving diagnostic bronchoscopy using the novel method of respiratory support, HFNC, in the intensive care unit.

## 2. Case Report

**Figure 1:**
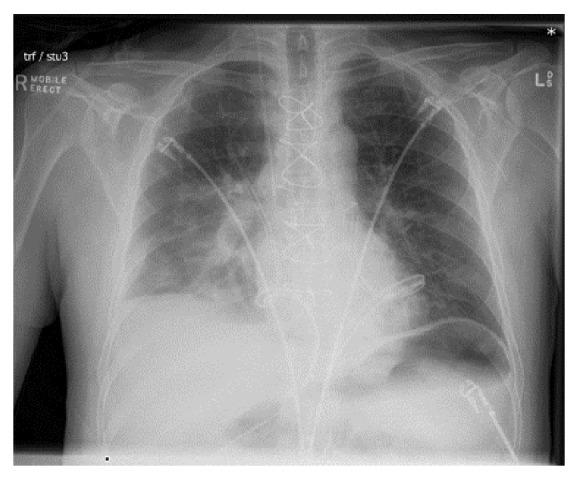
Prebronchoscopy chest X-ray.

A 62-year-old male received bilateral lung transplant for end stage idiopathic pulmonary fibrosis. Extubated two days post-transplant requiring 10 L/min of simple oxygen via a facemask to maintain PaO_2_ > 80 mmHg. Decreased breath sounds predominately on the right side were noted during chest auscultation on day two, worsening by day three with increasing respiratory rate, work of breathing, heart rate, and respiratory support. HFNC was initiated with 40 L/min flow and FiO_2_ 0.4. Believed to be largely mechanical, chest X-ray confirmed right middle and lower lobe collapse with elevated right hemidiaphragm, relative graft oversizing, and suspected sputum retention (Figure 1). Complementary to less invasive intervention including chest physiotherapy, mobilization, and nebulisers, diagnostic bronchoscopy was initiated to facilitate airway toilet to aid in improvement of pulmonary function and biopsy to ensure no acute rejection. We elected to use HFNC oxygenation for respiratory support during bronchoscopy with success. This case report has ethical clearance from the Research, Ethics and Governance Unit, The Prince Charles Hospital, Metro North Hospital and Health Service (AC/JL/Final Approval), and written and informed consent was obtained from the subject.

**Figure 2:**
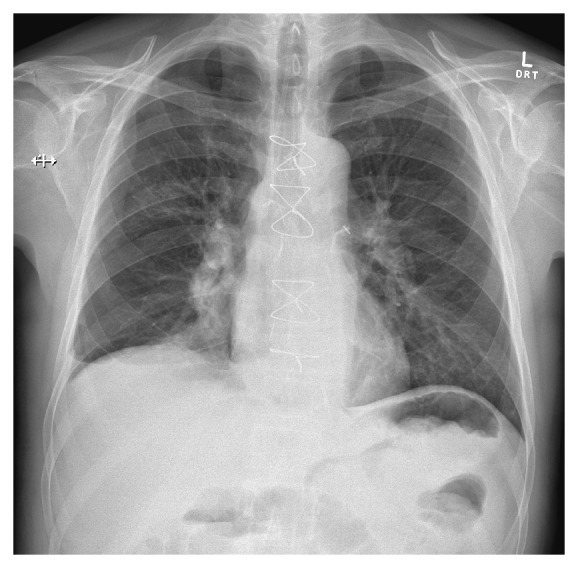
Postbronchoscopy chest X-ray.

Borderline candidate for reintubation for procedure with a PaO_2_/FiO_2_ ratio of 200. With improvements in respiratory rate and oxygen saturations after initiation on HFNC prior to bronchoscopy, HFNC support continued during local anaesthesia, intravenous sedation, and bronchoscopy with lavage and biopsy. After administration of sedation, 2.5 mg of midazolam and 100 mcg of fentanyl, FiO_2_ via HFNC were increased to 1.0 and flow remained at 40 L/min. Bronchoscopy showed significant secretion burden in the right intermediate bronchus and bilateral proximal airways, hyperaemia and edema of the right lower lobe basal segments, and secretions in the right middle lobe. Operator was able to perform extensive lavage freely to allow for airway patency as oxygen saturations remained at 100% during the procedure with no other complications. As subject woke, FiO_2_ was weaned to maintain oxygen saturations above 95%. HFNC was eventually weaned by day four and repeated chest X-ray showed significant improvement in lung fields (Figure 2). Subject was discharged from intensive care on day five.

## 3. Discussion

There are many theories on the mechanisms of action of HFNC. Numerous studies have demonstrated that HFNC produces pharyngeal pressures of 2–8 cm H_2_O and it is hypothesized that these pressures may contribute to lung recruitment and splinting open of upper airways. Additionally, HFNC has been shown to provide a dead space washout of the nasopharynx, and reduction of work of breathing by decreasing inspiratory resistance and by providing heated and humidified oxygen therapy, pulmonary compliance, and conductance are maintained [6–8]. Numerous studies have shown the safety and efficacy of HFNC in the acute clinical setting and it is proving to be promising multifunctional supportive modality [2, 4, 8–13].

Early complications associated with bronchoscopy can be severe [4, 14, 15]. In adults, the bronchoscope takes up to 10–15% of the tracheal lumen resulting in increased work of breathing and decreased PaO_2_ [14]. Respiratory support during bronchoscopy varies from topical local anesthetic with simple low flow nasal oxygen to general anesthesia with a laryngeal mask airway or endotracheal tube, maintaining spontaneous respirations. NIV strategies and their use during endoscopy have widely been investigated as a safe option for respiratory support [14]. Utilizing HFNC during bronchoscopy in this case report demonstrated an efficient intervention for maintaining oxygenation safely in an already compromised patient undergoing invasive procedure. HFNC during bronchoscopy could potentially reduce the need for general anesthetic in some patient groups. HFNC during bronchoscopy on sedated patients may serve as a worthy topic for future research.
